# Human bone marrow-derived stromal cell behavior when injected directly into the bone marrow of NOD-*scid*-gamma mice pre-conditioned with sub-lethal irradiation

**DOI:** 10.1186/s13287-021-02297-7

**Published:** 2021-04-12

**Authors:** Bianca Nowlan, Kathryn Futrega, Elizabeth Deborah Williams, Michael Robert Doran

**Affiliations:** 1 School of Biomedical Sciences, Faculty of Health, Queensland University of Technology (QUT), Brisbane, Australia; 2 Australian Prostate Cancer Research Centre – Queensland (APCCRC-Q) and Queensland Bladder Cancer Initiative (QBCI), Brisbane, Queensland Australia; 3 Translational Research Institute, 37 Kent Street, Brisbane, Queensland 4102 Australia; 4 Centre for Biomedical Technologies (CBT) and School of Mechanical, Medical, and Process Engineering (MMPE), Queensland University of Technology (QUT), Brisbane, Australia; 5 Skeletal Biology Section, National Institute of Dental and Craniofacial Research, National Institutes of Health, Building 30, 30 Convent Dr MSC 4320, Bethesda, MD 20892-4320 USA; 6 Mater Research Institute – University of Queensland, Brisbane, Australia; 7 Australian National Centre for the Public Awareness of Science, Australian National University, Canberra, Australia

**Keywords:** Sub-lethal irradiation, Xenograft, Intrafemoral injection, Human bone marrow-derived stromal cells, Bone marrow, Cell competition

## Abstract

**Background:**

Direct bone marrow injection of cells into murine marrow cavities is used in a range of cell characterization assays and to develop disease models. While human bone marrow-derived stromal cells (hBMSC, also known as mesenchymal stem cells (MSC)) are frequently described in therapeutic applications, or disease modeling, their behavior following direct injection into murine bone marrow is poorly characterized. Herein, we characterized hBMSC engraftment and persistence within the bone marrow of NOD-*scid* interleukin (IL)-2γ^−/−^ (NSG) mice with or without prior 2 Gy total-body γ-irradiation of recipient mice.

**Methods:**

One day after conditioning NSG mice with sublethal irradiation, 5 × 10^5^ luciferase (Luc) and green fluorescent protein (GFP)-expressing hBMSC (hBMSC-Luc/GFP) were injected into the right femurs of animals. hBMSC-Luc/GFP were tracked in live animals using IVIS imaging, and histology was used to further characterize hBMSC location and behavior in tissues.

**Results:**

hBMSC-Luc/GFP number within injected marrow cavities declined rapidly over 4 weeks, but prior irradiation of animals delayed this decline. At 4 weeks, hBMSC-Luc/GFP colonized injected marrow cavities and distal marrow cavities at rates of 2.5 ± 2.2% and 1.7 ± 1.9% of total marrow nucleated cells, respectively in both irradiated and non-irradiated mice. In distal marrow cavities,  hBMSC were not uniformly distributed and appeared to be co-localized in clusters, with the majority found in the endosteal region.

**Conclusions:**

While significant numbers of hBMSC-Luc/GFP could be deposited into the mouse bone marrow via direct bone marrow injection, IVIS imaging indicated that the number of hBMSC-Luc/GFP in that bone marrow cavity declined with time. Irradiation of mice prior to transplant only delayed the rate of hBMSC-Luc/GFP population decline in injected femurs. Clusters of hBMSC-Luc/GFP were observed in the histology of distal marrow cavities, suggesting that some transplanted cells actively homed to distal marrow cavities. Individual cell clusters may have arisen from discrete clones that homed to the marrow, and then underwent modest proliferation. The transient high-density population of hBMSC within the injected femur, or the longer-term low-density population of hBMSC in distal marrow cavities, offers useful models for studying disease or regenerative processes. Experimental designs should consider how relative hBMSC distribution and local hBMSC densities evolve over time.

**Supplementary Information:**

The online version contains supplementary material available at 10.1186/s13287-021-02297-7.

## Introduction

The mouse is a common biomedical model organism. Direct bone marrow injection of cells into the marrow cavity of mice is commonly used to study hematopoietic stem progenitor cell (HSPC) transplantation [1–5], or to study the behavior of cancer cells [6, 7]. While bone marrow-derived stromal cells (BMSC, also known as “mesenchymal stem cells”) are viewed as a critical component of the bone marrow microenvironment and are known to have a direct impact on HSPC engraftment or in cancer metastasis [8–10], it is not well understood how human BMSC (hBMSC) behave when directly injected into a murine marrow cavity. In this study, we sought to characterize how hBMSC behave when transplanted directly into the marrow cavities of mice that either had or had not been pre-conditioned with sublethal irradiation.

Few studies have explored the different routes of hBMSC injection in immune-compromised mice and pre-conditioning of these animals with irradiation. When hBMSC were transplanted via intravenous injection into immune-compromised mice conditioned with sublethal irradiation, hBMSC could be found in tissues such as the spleen, muscles, liver, lungs, and bone marrow [11]. Another study demonstrated that irradiation of a localized tissue site induced homing of hBMSC, as well as promoted their widespread engraftment into multiple organs distal from the localized irradiation [12]. In a model where both hBMSC and human HSPC were co-transplanted via direct bone marrow injection, HSPC engraftment was enhanced by the inclusion of hBMSC [2]. While human hematopoietic cell number increased in the animals’ bone marrow and peripheral blood between the 3**-**week and 6-week time points following HSPC transplantation, hBMSC numbers in the injected marrow cavity approximately halved over the 6-week period. Unlike the previous studies [11, 12], no hBMSC were found in distal marrow cavities where hBMSC were not directly injected [2]. In a control arm of the study, hBMSC were transplanted via intravenous injection, but no hBMSC were found in the marrow cavities at the end of the 6-week study. All three studies used NOD-*scid* mice with conditioning regimens - specifically 3 Gy irradiation [2], 3.5 Gy irradiation [11], or irradiation ranging from 3.5–4.5 Gy total body or 26.5 Gy in localized areas [12].

Since the publication of the studies described above, the NOD-*scid* interleukin (IL)-2γ^−/−^ (NSG) mouse has replaced the NOD-*scid* mouse as the most commonly used immune-compromised mouse model [13]. Unlike the NOD-*scid* mouse model, the NSG mouse does not have natural killer cells, making it more tolerant of xenografts [14]. The NSG mouse is routinely used to study human HSPC engraftment. While the NSG bone marrow microenvironment is permissible to human HSPC engraftment without prior conditioning with irradiation, conditioning animals prior to transplant does significantly improve engraftment [14]. For example, 12 weeks after human HSPC transplant, human CD45 cell content in peripheral blood increased from ~ 5% in non-conditioned mice to ~ 60% in mice that had been conditioned with 2.5 Gy irradiation [15]. In previous studies completed in our laboratory, 2 Gy conditioning of NSG mice was sufficient to facilitate human CD45 engraftment rates as high as 95% within the bone marrow cavities of femurs that had been directly injected with human HSPC 8 weeks earlier [1].

The capacity to establish and maintain a significant population of hBMSC within the bone marrow cavity of the NSG mouse has not been fully characterized. Herein, we compared the capacity to populate NSG mouse marrows with hBMSC in mice that either had or had not been conditioned with 2 Gy irradiation prior to hBMSC transplant via direct bone marrow injection. To facilitate tracking, hBMSC were transduced to constitutively express the luciferase enzyme (Luc) and green fluorescent protein (GFP) (hBMSC-Luc/GFP). NSG mice each received 5 × 10^5^ hBMSC-Luc/GFP, transplanted via intrafemoral injection into the right femur. The persistence of transplanted hBMSC-Luc/GFP was quantified using bioluminescence and histology, while mouse hematopoietic recovery following irradiation was characterized via flow cytometry.

## Methods

### Human bone marrow collection and hBMSC expansion

The collection and use of human bone marrow were approved by the Mater Hospital Human Research Ethics Committee and by the Queensland University of Technology (QUT) Human Research Ethics Committee (Ethics No.: 1000000938). All donors provided informed voluntary written consent, and all processes followed the National Health and Medical Research Council of Australia guidelines. hBMSC were derived from the bone marrow aspirates from two healthy male donors (donor 1: 40–49-year-old male; donor 2: 20–29-year-old male; exact ages withheld to protect the identity of the donors) using previously described protocols [16]. Cells were isolated by plastic adhesion and expanded in growth medium containing low-glucose Dulbecco’s modified Eagle’s medium (DMEM), 10% fetal bovine serum (FBS, Gibco, (Catalog number F1917, Lot 1652793)), and 10 ng/mL fibroblast growth factor-1 (FGF-1, Peprotech), in a humidified 2% O_2_ and 5% CO_2_ incubator. Our group has optimized these culture conditions over time and reported on them previously [16–18]. Both hypoxia [19] and FGF media supplementation [20] are known to facilitate BMSC maintenance in culture.

### Transduction of hBMSC

A third-generation lentiviral system was used to transduced hBMSC to express luciferase/GFP, driven by a Murine Stem Cell Virus promotor (MSCV, System Bioscience, pBLIV301PA-1). The MSCV promotor was chosen due to effectiveness in stem cell populations, reliability through differentiation, and as it is rarely silenced once in the mouse [21–23]. Lentiviral particles were manufactured using HEK293T cells transfected with a TGEN packaging plasmid mix using a 1:3 ratio of plasmid in reagent (μg DNA: μL Lipofectamine 2000; Thermo Fisher Scientific). Virus containing conditioned medium from the HEK293T cells was harvested and used to transduce early passage hBMSC. Three days after exposure of hBMSC to viral particles, GFP^+^ hBMSC were selected by flow sorting (Beckman Coulter Astrios), and then further expanded in culture. Expanded hBMSC-Luc/GFP were frozen at passage 3–4 and thawed for use as needed. All subsequent experiments used passage 4–6 hBMSC-Luc/GFP.

### In vitro characterization of hBMSC

At passage 1, prior to transduction, hBMSC were characterized for cell surface markers CD105, CD90, CD73, CD146, CD44, CD45, CD34, CD31, and HLA-DR, using flow cytometry. Following stable transduction, passage 4 hBMSC-Luc/GFP were characterized for the retention of mesenchymal markers CD105, CD90, CD73, CD44, and CD146, the absence of the hematopoietic marker CD45, and the presence of a GFP signal. All antibodies are listed in Supplementary Table 1. Antibody staining was performed as recommended by the manufacturers’ instructions. Flow cytometry was performed on a Becton Dickinson (BD) Fortessa or a Beckman Coulter Cytoflex, and data analyzed using FlowJo Software (BD). The capacity of hBMSC-Luc/GFP to undergo adipogenic and osteogenic differentiation was assessed in monolayer culture. hBMSC were seeded (1.2 × 10^5^) in monolayer culture in 48-well plates in either adipogenic or osteogenic induction medium. Chondrogenesis was evaluated using pellet cultures formed from 2 × 10^5^ hBMSC-Luc/GFP each. Adipogenesis was evaluated using Oil Red O staining for lipid vacuoles, osteogenesis using Alizarin Red S staining for mineral formation, and chondrogenesis using Alcian Blue staining for glycosaminoglycans (GAG). The medium formulations and methods used for tri-lineage differentiation have been previously described [16, 18, 24]. While results from these assays are qualitative in nature, we routinely perform 4 technical replicates for each of the osteogenic, adipogenic, and chondrogenic cultures.

To test for luciferase activity in hBMSC-Luc/GFP, the cells were seeded into 96-well black tissue culture plates (Corning) at 2 × 10^4^ cells per well in duplicate and titrated using a repetitive 1:1 serial dilution and allowed to attach in an incubator overnight. The following day, media was exchanged with pre-warmed XenoLight D-luciferin (Perkin Elmer, Cat No. 122799), diluted in growth medium at 150 μg/mL. Bioluminescence was measured immediately using a Spectrum In Vivo Imaging System (IVIS, Perkin Elmer) and then analyzed using LiveImage Software (Perkin Elmer).

### Direct bone marrow transplantation of hBMSC into mice

Mouse experimental procedures were approved by the University of Queensland Animal Ethics Committee and by the QUT Ethics Committee (Ethics No.: 1500000055 and 1600000951). All animal work was performed following guidelines set out by the National Health and Medical Research Council of Australia. NSG mice were purchased from Jackson Laboratories (Bar Harbour, ME; Stock No. 001976) and bred at the Translational Research Institute Biological Research Facility (Brisbane, Australia). Male and female mice were 8-weeks old were used in experiments. We aimed to balance the gender composition of groups, but otherwise assigned mice randomly to two groups. One group of mice received 2 Gy of sub-lethal total body irradiation (^137^Cs, Gammacell 40 Exactor, Best Theratronics, Ontario, Canada) 24 h prior to hBMSC-Luc/GFP transplantation, while the other group received no irradiation. hBMSC-Luc/GFP (5 × 10^5^) cells suspended in 10 μL of X-VIVO 15 medium (Lonza), were injected into the right femur of mice using a Hamilton syringe as our previously described protocol [1]. On the day of hBMSC-Luc/GFP injection, and every week post-transplant, mice were administered an intraperitoneal injection of D-luciferin (150 mg/kg body weight), and 10 min later animals were imaged for bioluminescence using an IVIS [25], and analyzed using LiveImage software. If a bioluminescence signal was detected in the lung on the day of hBMSC injection, this was taken to indicate that the transplant of the hBMSC-Luc/GFP into the marrow cavity was unsuccessful, and that mouse was excluded from subsequent analysis.

### Histology

Mouse legs, lung, liver, heart, and spleen were collected at 4-weeks post-hBMSC-Luc/GFP transplant for histology. At week 1, injected femurs were also harvested for histology. Bone and organ samples were fixed in 4% paraformaldehyde (PFA, Sigma-Aldrich) in phosphate-buffered saline (PBS) solution. Femurs and tibias were decalcified in a solution of 15% ethylenediaminetetraacetic acid (EDTA) and 0.5% paraformaldehyde (PFA) in PBS and then embedded in paraffin. A microtome (Leica, Cat No. RM2235) was used to create 5 μm tissue sections, which were adhered to Superfrost Plus slides (Thermo Fisher Scientific). Antigen retrieval was performed (10 mM sodium citrate, 0.05% tween 20, pH 6.0, for 20 min at 95 °C), followed by blocking with Background Sniper (BioCare Medical, Cat No. BS966), and then stained overnight at 4 °C with primary antibodies. Sections were washed and stained with secondary antibodies for 2 h, washed and counterstained for 10 min with 1 μg/mL 4′, 6-diamidino-2-phenylindole (DAPI, Thermo Fisher Scientific) for nuclei identification, and then coverslipped with Prolong Gold (Thermo Fisher Scientific). All antibodies and concentrations used in this project are listed in Supplementary Table 1.

To identify cycling hBMSC-Luc/GFP, tissues were stained with anti-Ki-67 antibody (Roche). To identify apoptotic hBMSC-Luc/GFP in tissue sections, terminal deoxynucleotidyl transferase dUTP nick end labeling (TUNEL) was performed using the In Situ Cell Death Detection Kit, TMR red (Roche). Tissues were subsequently stained with anti-GFP antibody and DAPI to show colocalization of TUNEL or Ki-67 with hBMSC-Luc/GFP.

Slides were imaged on a 3DHistech Slide Scanner at × 20 magnification. Resultant images were analyzed on the Case Viewer software (V2.2, 3DHistech) and staining quantified using ImageJ [26]. Slides were imaged using autofocus and auto acquisition protocol. Background fluorescence was quantified by scanning an unused channel, and these data were used to threshold the sample.

Six-to-nine × 20 randomly selected images were taken of the bone marrow of each animal and used to estimate the number of hBMSC-Luc/GFP, relative to total nucleated cell bone marrow content. Images were randomly selected, using a random number generator, from histology sections that had been scanned. However, in some instances, this approach selected a region on the slide that was not usuable because either the tissue was damaged in that location, or because of imperfections in the machine’s autofocus. In these instances, another area was randomly selected so that there were sufficient replica input images for the analysis. Histology samples were counted while masked by mouse number and only later grouped after analysis had been completed. The location of hBMSC-Luc/GFP was analyzed in relation to marrow sinusoids, blood vessels, and relative distance from the bone or to other hBMSC-Luc/GFP. In our analysis, the endosteal region was defined based on previous publications [27, 28] to be ~ 13 cell diameters from the bone equating to 70 μm in this study. This accounted for approximately 20% of the whole bone marrow cavity which measured ~ 700 μm across. To quantify cycling or apoptotic hBMSC-Luc/GFP, three 40X GFP^+^-rich fields were acquired. Proliferating (Ki-67^+^) or apoptotic (TUNEL^+^) cells were counted as a frequency of DAPI^+^ hBMSC-Luc/GFP cells. All replicates were averaged, and then treatment groups were compared.

### Characterizing hematopoietic recovery following irradiation

To estimate the recovery of the bone marrow, we repeated our experimental study where mice were subjected to hBMSC-Luc/GFP intrafemoral transplant following 2 Gy irradiation or no irradiation. Tail bleeds were performed before transplant and at day 3, 7, 10, 14, and 21 following the transplantation of hBMSC-Luc/GFP. A complete blood count (white blood cells, red blood cells, hemoglobin, and platelets) was performed using a Coulter AcT Diff Analyzer (Beckman Coulter), then red blood cells lysed (as previously described [29]) and further characterization performed using flow cytometry (antibodies listed in Supplementary Table 1).

### Statistics

All statistical analysis was completed using Graph Pad Prism 8 (La Jolla, CA). Data was tested with Shapiro-Wilk and the Kolmogorov-Smirnov normality tests before analysis. Repeated measure data was analyzed using linear regression with the Akaike’s Information Criterion (AICc) fit test used to assess model appropriateness. Between groups, statistical analysis was performed using multiple *t* test, 2-way ANOVA, or mixed-effect model with multiple comparison utilizing the Holm-Sidak’s correction as indicated in the figure legend. A comparison of data to theoretical values was completed using a one-sample *t* test. *P* < 0.05 was considered statistically significant.

## Results

### In vitro characterization of hBMSC

Cells isolated from both hBMSC donors were found to be > 95% positive for the mesenchymal stromal cell markers CD105, CD90, CD73, CD146, and CD44; negative for hematopoietic markers CD45, CD34, and HLA-DR; and negative for the endothelial marker CD31 (Supplementary Figure 1A and 1C). These cells demonstrated expected mesodermal differentiation capacity, forming lipid vacuoles in adipogenic induction medium, a bone-like matrix in osteogenic induction medium, and a cartilage-like matrix in the chondrogenic induction medium (Supplementary Figure 1B and D).

### In vitro characterization of hBMSC-Luc/GFP

Transduced hBMSC-Luc/GFP maintained tri-lineage differentiation potential (Supplementary Figures 2C-E, 3C-E) and maintained stable expression of GFP, which was validated by fluorescent microscopy and flow cytometry over time and cell passaging (GFP^+^ cells, Supplementary Figures 2B, and F, 3B and F). Following transduction, hBMSC-Luc/GFP continued to be > 95% positive for mesenchymal stromal cell markers CD105, CD90, CD73, CD146, and CD44 and negative for CD45 (Supplementary Figures 2F and 3F). Titrations of hBMSC-Luc/GFP were used to demonstrate that the bioluminescence signal increased linearly with hBMSC-Luc/GFP number in the presence of D-luciferin (Supplementary Figures 2G, and H; 3G, and H).

### Tracking hBMSC in live animals with bioluminescence

**Fig. 1:**
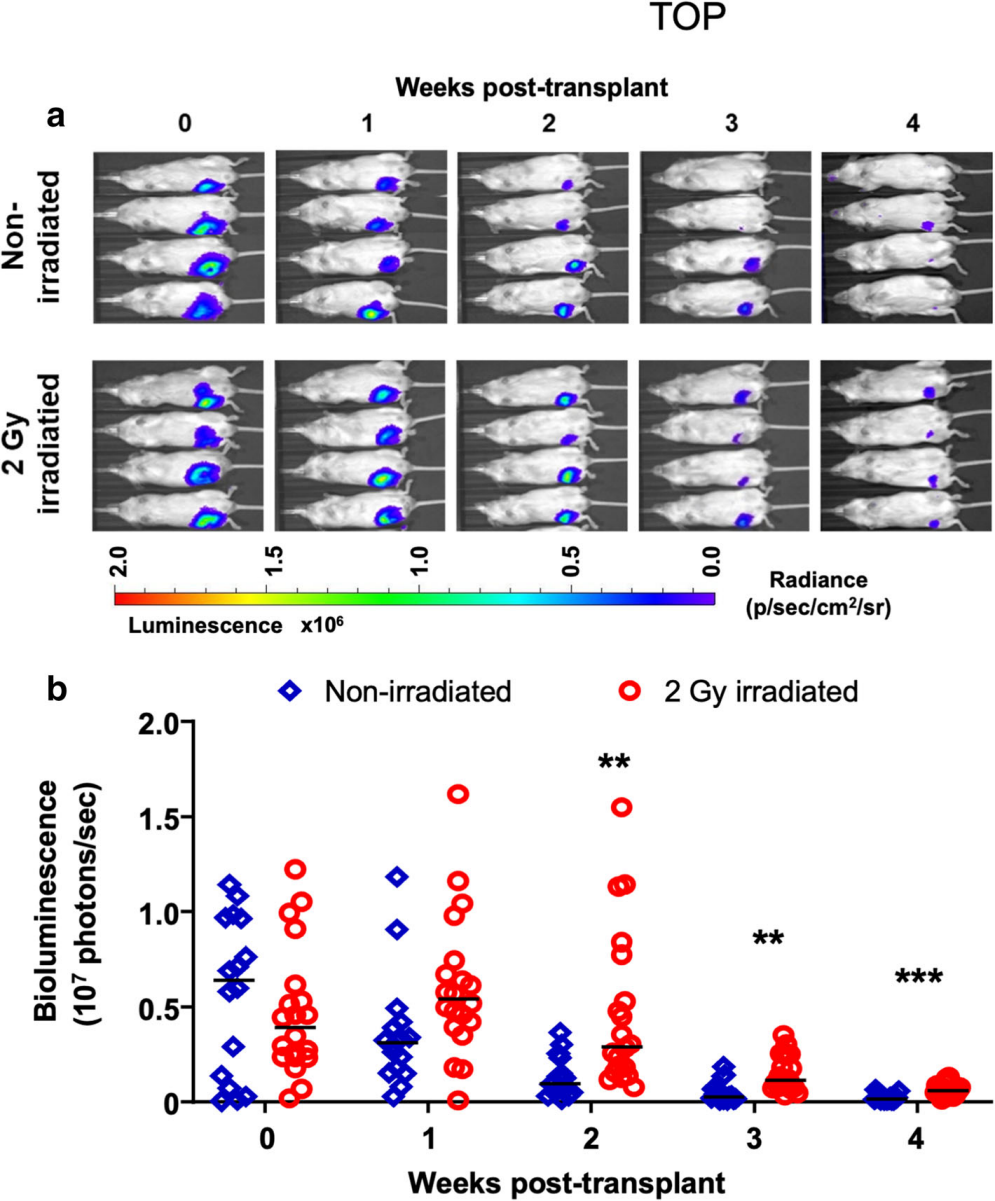
Comparison of hBMSC-Luc/GFP persistence in the femurs of mice with and without 2 Gy sub-lethal irradiation prior to transplant. **a** Representative IVIS images of the bioluminescence signal from mice that had either been non-irradiated or had been conditioned with 2 Gy irradiation. **b** Graphical display of the bioluminescence data for each animal across the 4-week study. Horizontal bar represents the group average. Linear regression curves were fitted to each data set. The signal in non-irradiated mice was significantly lower than in irradiated animals from week 2 to week 4 (2-way ANOVA with multiple comparison using Holm-Sidak correction). Significance between groups marked with asterisk; ** *p* ≤ 0.01, *** *p* ≤ 0.001. Data represents pooled data the two donor hBSMC and each with a biological repeat (total group *n* = 16 mice not irradiated, and *n* = 20 mice received 2 Gy irradiation). Donors are plotted separately in Supplementary Figure 4A. The graphical display in Supplementary Figure 4B shows the data fit by independent regression lines for irradiated and non-irradiated mice. Akaike’s Information Criterion (AICc) test in GraphPad was used to estimate the probability (99.92%) that the irradiated and non-irradiated mouse data was better fit by two independent curves, than a single curve, suggesting that BMSC behavior was statistically different in irradiated and non-irradiated mice

Mice were conditioned with or without 2 Gy sub-lethal irradiation the day prior to transplantation. Mice received injections into the right femur with hBMSC-Luc/GFP or media only. This experiment was repeated with the two unique hBMSC-Luc/GFP donors to account for donor heterogeneity. Bioluminescence signal from the hBMSC-Luc/GFP was restricted to the transplanted femur, with no bioluminescence signal detected elsewhere (Fig. 1a). Here we found the two hBMSC donors behaved similarly in the mouse model (Supplementary Figure 4A). In non-irradiated mice, femur bioluminescence signal decreased from the first week after transplant, while in irradiated mice, a stable signal was maintained until week 2 after transplant (Fig. 1b). The bioluminescence signal in non-irradiated mice was significantly lower than in irradiated animals from week 2 to week 4 (multiple *t* tests with Holm-Sidak correction method). By week 4, the bioluminescence signal from all animals had declined significantly. Regression analysis was used to fit curves to the data from all non-irradiated and all irradiated animals (Supplementary Figure 4B). Using Akaike’s Information Criterion (AICc) test in GraphPad, it was predicted that it was probable (99.92%) that the data from the irradiated and non-irradiated mice was better explained two independent curves, rather than a single curve. This analysis suggests that hBMSC behavior was statistically different in irradiated and non-irradiated animals. These data were replotted again in Supplementary Figure 5 to show outcomes in male and female mice. No gender-associated differences were observed.

### Histological characterization of transplanted mice

**Fig. 2:**
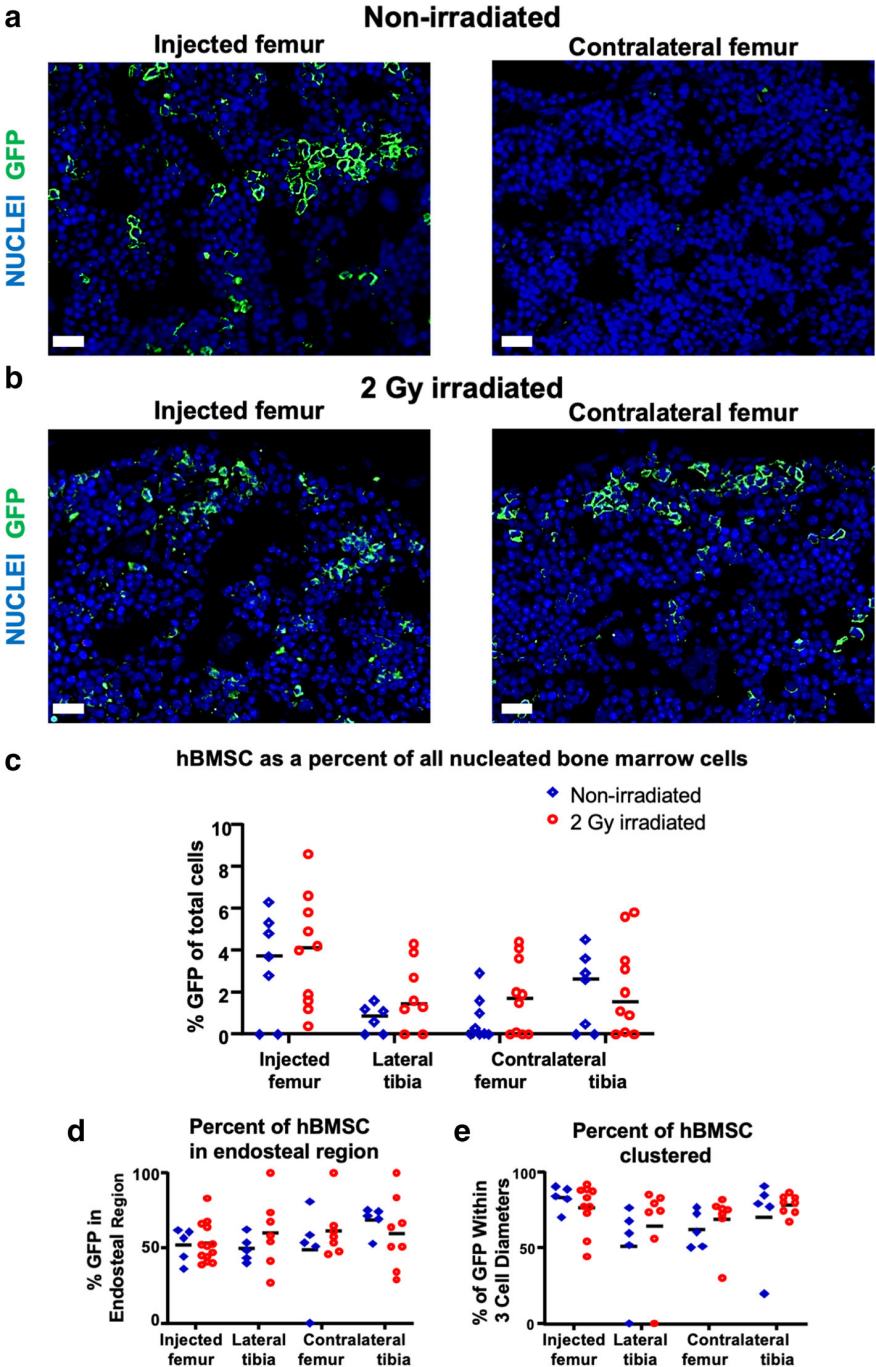
hBMSC-Luc/GFP engraftment in **a** Non-irradiated and **b** 2 Gy Irradiated mice detected by immunohistochemistical staining of mouse injected and contralateral femurs at week 4, post-transplant. Scale bar equals 20 μm. **c** The percentage of hBMSC-Luc/GFP detected in the bone marrow cavities of non-irradiated (blue) or irradiated mice (red). **d** The distribution of hBMSC-Luc/GFP in relation to the endosteal region. **e** The proportion of hBMSC-Luc/GFP that are within 3 cell diameters of another hBMSC-Luc/GFP. Each data point represents a single mouse, and the horizontal bar represents the group average. For some animals, we were unable to capture sufficiently high-quality histology images, and in these instances, histology data for these animals is not reported. High-quality histology was captured for *n* = 8 non-irradiated mice, and *n* = 10 irradiated mice. Significance was tested using 2-way ANOVA with the Holm-Sidak’s multiple comparison test between groups

Histology was used to quantify the number of hBMSC-Luc/GFP in the bone marrow where the bioluminescent signal was too weak to be quantified using the IVIS. An anti-GFP antibody was used to detect and amplify hBMSC-Luc/GFP in histological sections of injected femurs, both tibias, and non-injected contralateral femurs in non-irradiated and 2 Gy irradiated mice (Supplementary Figure 6). Bone marrow cavities in mice that had not been transplanted with hBMSC-Luc/GFP had no GFP^+^ cells (Supplementary Figure 6B). The number of GFP^+^ cells in injected femurs was greater than in non-injected contralateral femurs, but did not reach significance at this late time-point (Fig. 2a-c). Similarly, the differences between the treatment groups were not significant at the 4-week harvest. Injected femurs at 4 weeks contained 3.6 ± 0.5% hBMSC-Luc/GFP while the dissemination of cells discovered in the contralateral femurs and tibias yielded 1.7 ± 1.8% hBMSC-Luc/GFP of all nucleated cells in the bone marrow. Detailed numbers and analysis are presented in Supplementary Table 2.

We found no specific pattern of distribution for hBMSC-Luc/GFP within the sinusoidal or perivascular hematopoietic stem cell niches (Supplementary Figure 8A-B). However, hBMSC-Luc/GFP were most commonly identified within the endosteal region, relative to the rest of the bone marrow volume (Fig. 2d). We averaged data captured from replicate images to estimate the number of hBMSC-Luc/GFP in the endosteal region for 18 mice. These data indicated a frequency of hBMSC-Luc/GFP in the endosteal region of 54.5 ± 12.4%, compared to the theoretical random distribution within that bone fraction being 20% (Supplementary Figure 7C). Using a one sample *t* test (*t* (20) = 12.74, *p* = < 0.0001), this distribution of hBMSC-Luc/GFP in the endosteal region was found to be statistically significantly different to random distribution. The majority of hBMSC-Luc/GFP in distal marrow cavities were found to be clustered within 3 cell-diameters of each other (Fig. 2e). hBMSC-Luc/GFP were not detected in the lungs, livers, hearts, or spleens at the 4-week timepoint (data not shown).

### Murine hematopoietic recovery following irradiation

**Fig. 3:**
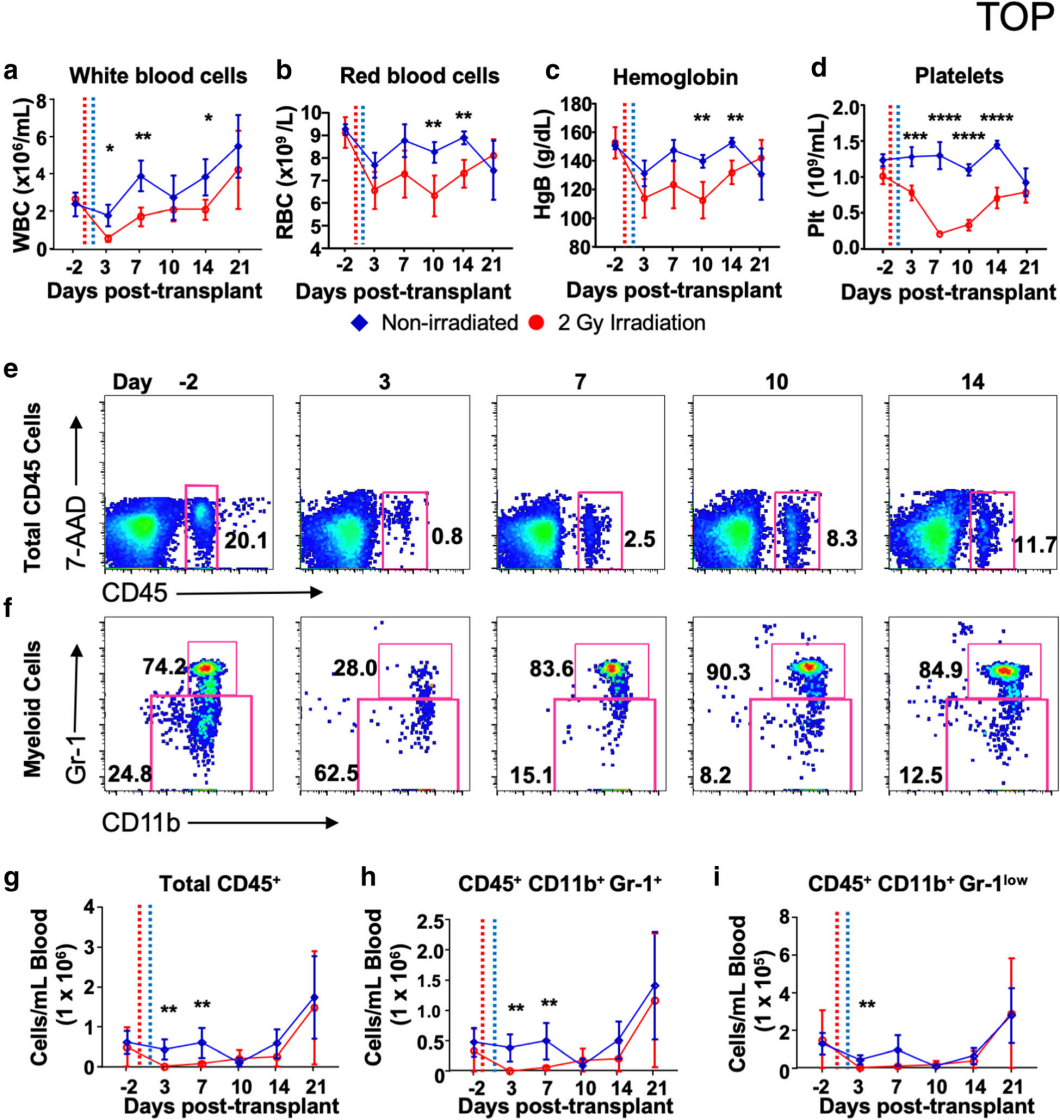
Characterization of murine hematopoietic cell recovery following sub-lethal irradiation. **a** Total white blood cells, **b** red blood cells, **c** hemoglobin, and **d** platelet levels overtime are displayed graphically. Irradiation treatment is denoted on the graphs with a dotted red line, and the hBMSC transplant denoted by a dotted blue line. Error bars represent the group standard deviation. **e** Dot plots of blood cells (CD45^+^ 7-AAD^−^) can be seen over time in the irradiated group, revealing the decline at day 3 and recovery by day 10. **f** Eosinophils, neutrophils, classical monocytes, and macrophages (CD11b^+^, Gr-1^+^), and non-classical monocytes (CD11b^+^, Gr-1^low^) also show the loss and recovery of the cells over time in the irradiated group. **g**–**i** Graphical representation of recovery. Data were analyzed with multiple *t* tests with Holm-Sidak’s correction and a 0.95% confidence level significance indicated by asterisk * *p* ≤ 0.05, ** *p* ≤ 0.01, *** p ≤ 0.001, **** *p* ≤ 0.0001. Groups non-irradiated *n* = 4, irradiated *n* = 6

We quantified the recovery of murine hematopoietic cell populations following sub-lethal irradiation, and hBMSC-Luc/GFP intrafemoral transplant (Fig. 3). As expected, blood cell count parameters declined following irradiation (Fig. 3a–d). White blood cell numbers recovered to control levels by day 10, while red blood cells, hemoglobin, and platelet levels recovered by day 21. Red blood cell and platelet numbers did not decline to clinical anemic or thrombocytopenic levels, respectively [30].

Blood was lysed to eliminate red blood cells, and the remaining cells were stained with fluorescence-conjugated antibodies against mouse CD45, CD11b, and Gr-1 and with live/dead cell discriminator 7-AAD and analyzed by flow cytometry. Eosinophils, neutrophils, classical monocytes, and the rare blood macrophage population are defined as CD45^+^, CD11b^+^, and Gr-1^+^, while non-classical monocytes were defined as CD45^+^, CD11b^+^, and Gr-1^low^ [31]. Total live mouse CD45^+^ cells in mice were depleted significantly at day 3, becoming leukopenic (< 1500 cells/μL, Fig. 3e, g). Myeloid cells were similarly depleted by day 3, with Gr-1^+^ cells predominately made up of neutrophils dropped to neutropenic levels (10 cells/μL, Fig. 3f, h, i) [30]. Non-classical monocyte numbers also decreased in the irradiated group and recovered by day 10. Blood parameters are further detailed in Supplementary Table 3.

### Proportion of hBMSC progressing through cell division

**Fig. 4:**
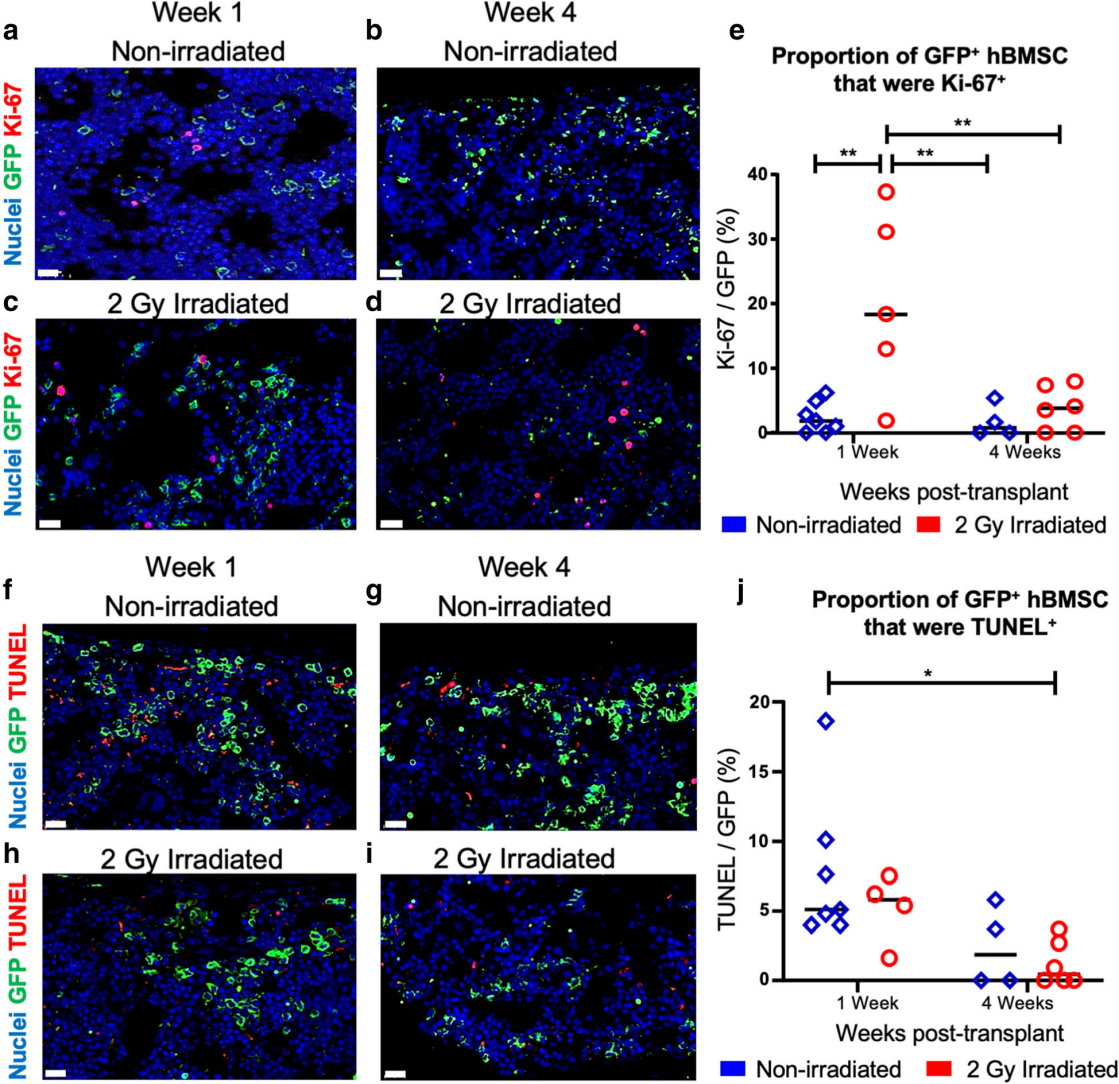
Cell cycle and apoptosis analysis. Images of injected femur bone marrow stained for GFP (greed), DAPI (blue), and Ki-67 (red) at week 1 and week 4 in non-irradiated (**a**, **b**) and irradiated mice (**c**, **d**). Scale bars = 20 μm. **e** Quantification of Ki-67^+^ hBMSC from non-irradiated animals (blue, week 1 *n* = 7, week 4 n = 4), and 2 Gy irradiated animals (red, week 1 n = 7, week 4 n = 6). Horizontal bars represent averages, each point represents an individual mouse. Image of femur bone marrow from stained for GFP (green), DAPI (blue), and TUNEL (red) at week 1 and week 4 in non-irradiated (**f**, **g**) and irradiated mice (**h**, **i**). Scale bar = 20 μm. **j** Quantitative analysis of TUNEL staining in hBMSC-Luc/GFP (non-irradiated animals are represented in blue (week 1 *n* = 7, week 4 *n* = 4) and 2 Gy irradiated animals are in red (week 1 *n* = 7, week 4 *n* = 6). Horizontal bars represent averages, each point represents an individual mouse. Statistical significance calculated by two-way ANOVA with Holm-Sidak’s multiple correction using a 95% confidence level, significance identified by asterisk, * *p* ≤ 0.05. * p ≤ 0.05, ** *p* ≤ 0.01

Bone marrow histological sections were stained for Ki-67, an active cell cycling marker, to estimate the proportion of hBMSC-Luc/GFP which were progressing through cell division (Fig. 4a, b). The faction of Ki-67^+^ hBMSC-Luc/GFP was greater in irradiated mice, relative to the non-irradiated mice, at week 1 post-transplant. By week 4, irradiated and non-irradiated mice had similar proportions of Ki-67^+^ hBMSC-Luc/GFP. No change in the number of cycling cells was detected in the non-irradiated group with time. Thus, a greater portion of the hBMSC-Luc/GFP in the irradiated femurs were actively cycling following transplant, potentially contributing to greater maintenance or possible expansion of this population at week 1, relative to non-irradiated animals.

### Proportion of hBMSC undergoing apoptosis in mouse bone marrow

Using TUNEL staining, an indicator of late apoptosis [32], we estimated the percentage of hBMSC-Luc/GFP undergoing apoptosis in injected femurs at weeks 1 and 4 (Fig. 4f–h). Overall, few hBMSC-Luc/GFP were undergoing apoptosis as detected by TUNEL. There was a trend toward a greater rate of apoptosis in non-irradiated week 1 femurs, but this was not statistically significant between treatments at the same time-points. Similarly, the difference in TUNEL staining from week 1 to week 4 was not statistically significant within the same treatment group.

## Discussion

The mouse is a common experimental and pre-clinical research model organism, and understanding how human cells behave in the mouse is critical to advancing research and medicine. Direct injection of human HSPC into murine bone marrow is commonly used to study transplantation and engraftment [1–5]. Similarly, direct bone marrow injection of human cancer cells is routinely used to study metastatic cancer [6, 7]. While the murine bone marrow provides an excellent model system, not all murine components directly cross-react with the human cells used in these xenograft models [33, 34], and for this reason, there is merit in considering efforts to humanize aspects of the murine marrow compartment. The bone marrow compartment is a unique environment where, as described by Paolo Bianco, the bone marrow stem cell niches “bring into focus the cross-regulation of skeletal and hematopoietic physiology as rooted into the interplay of two stem cells (hematopoietic and skeletal) sharing a single niche [35].” Theoretically, augmenting mouse bone marrow with both human hematopoietic and skeletal cells would result in the mouse being a better model of human biology and disease. While many studies have characterized the behavior of human hematopoietic cells in the mouse bone marrow, and demonstrate the feasibility of engrafting human hematopoietic stem cells into mice [1, 14, 15, 18], few studies [11, 12] have only modestly characterized the behavior of human skeletal stem cells in the mouse bone marrow.

Herein we studied direct bone marrow injection of hBMSC into the femurs of NSG mice. Using live animal imaging we tracked the bioluminescent signal from transplanted hBMSC. While the signal from hBMSC injected into femurs was initially strong, this signal tapered rapidly over 4 weeks. Irradiation of animals prior to transplantation led to greater persistence of the signal from the injected hBMSC over the 2 weeks immediately following transplantation, but this signal also diminished significantly by 4 weeks.

Since we completed this experimental work, a publication by Bunting et al. characterized the leakage of cells directly injected into the femurs or tibias of mice [36]. For NSG mouse femurs they concluded that leakage from the bone marrow cavity occurred even if the injection volume was as little as 3 μL. It is common for studies to inject volumes of 10 μL [1, 37, 38], but sometimes injection volumes are greater (up to 40 μL [39–42]). The injected marrow cavity acts as a sieve retaining many cells, while some cells are lost to the general circulation. We injected a 10 μL cell suspension into femurs. The immediate bioluminescence signal from the femurs indicated that a significant population of cells were retained within the injected marrow cavity. We excluded mice demonstrating significant bioluminescence signal from the lungs on day 0 as this is evidence that there was significant dissemination of hBMSC outside of the femur. When animals were euthanized, histology revealed the presence of hBMSC in distal marrow cavities of the lateral tibia and contralateral tibia and femur. We presume that these cells were lost to the animals’ circulation at the time of transplantation, and homed to other bone marrow cavities. While 100% retention within the injected marrow cavity is not possible, we recommend that others review the data presented by Bunting et al. [36], and consider reducing the injection volumes.

We observed that distal bone marrow in the lateral tibia and contralateral tibia and femur were populated with hBMSC at a rate of 1.7 ± 1.8% of nucleated bone marrow cells. A previous study used intravenous transplantation to delivered expanded Stro-1^+^ or Stro-1^−^ hBMSC into sub-lethally irradiated NOD-*scid* mice, and observed that hBMSC homed and persisted in the bone marrow [11]. They found 13 ± 7 and 6 ± 10 hBMSC per 10,000 murine cells in the bone marrow, for Stro-1^+^ or Stro-1^−^ hBMSC by genomic analysis, respectively. When these values are expressed as percentages to enable comparison with our data, the values are 0.13% Stro-1^+^ and 0.06% Stro-1^−^ hBMSC, respectively. Another study, which also used NOD-*scid* mice, detected hBMSC in the bone marrow at a frequency of 0.20% of cells in non-irradiated animals versus 0.35% of cells in irradiated animals [12]. The frequency of hBMSC in the marrow of mice in our study was approximately 5–10-fold greater than these two earlier reports. Likely the increased frequency observed in our study is associated with our use of NSG mice, which are more tolerant of xenografts than the NOD-*scid* mice [13, 14].

While hBMSC were frequently observed in the distal marrow cavities of mice, their distribution did not appear random, motivating us to look for patterns. More hBMSC were localized within the endosteal region close to the bone, rather than in the central marrow cavity. The bone marrow endosteal niche is where HSPC are maintained, and is where HSPC home and engraft into following transplantation [43]. Thus, the observed pattern of hBMSC distribution is consistent with this aspect of HSPC transplant biology. The majority of hBMSC in distal bone marrow cavities were found to be within 3 cell diameters of another hBMSC. As we did not perform lineage tracing, it is not possible to demonstrate that the clustered cells are clonally related. However, the propensity for hBMSC to appear in clusters is consistent with hBMSC homing to the marrow during transplant, and subsequently dividing to form localized clusters of cells which could be clonally related. A similar number of hBMSC in the distal marrow cavities of both irradiated and non-irradiated mice suggest that irradiation may not have modified homing or expansion, which is dissimilar to historical work [12]. As we injected hBMSC into the marrow of mice, the number of hBMSC introduced into the circulation was not standardized and so the relative number of hBMSC in distal marrow cavities of irradiated and non-irradiated animals should not be over-interpreted. These data do demonstrate, similar to previous human HSPC engraftment studies [15], that NSG mice do not need to be irradiated to achieve at least some level of engraftment.

Because irradiation appeared to influence hBMSC persistence within injected femurs, we characterized the kinetics of the NSG mouse hematopoietic recovery for 21 days following sub-lethal irradiation. While all blood cell parameters declined significantly within 3 days of irradiation, white blood cell counts recovered after 10 days, and red blood cells, hemoglobin, and platelet levels recovered by 21 days. These data indicate that NSG mouse hematopoiesis is briefly compromised by 2 Gy irradiation. Based on the blood cell recovery period, hBMSC likely had a brief proliferative advantage in the irradiated marrow. Given the significant hematopoietic recovery by day 10, we reason that any proliferative or competitive advantage that hBMSC would have had in the murine marrow would have tapered rapidly by this time. The bioluminescence signal from the hBMSC in irradiated mice began a rapid decline 2 weeks after transplantation, approximately aligning with the recovery of hematopoiesis in these animals. Thus, the short-term greater persistence of hBMSC-Luc/GFP in the injected marrow cavities of irradiated mice was likely related to transient competitiveness of the hBMSC-Luc/GFP relative to recovering murine marrow. Intertwined with the recovery of the murine marrow, could be the depletion of transplanted hBMSC *so-called* clonal potential [44, 45] or possibly a monocyte/macrophage-mediated clearing of the hBMSC [46]. While an immune cell-mediated hBMSC clearing process may be attractive, we reason that the abundance of hBMSC within the distal marrow cavities of non-irradiated animals suggests this is unlikely the mechanism underpinning hBMSC depletion within injected femurs. In healthy adult human marrow, hBMSC make up ~ 0.001–0.01% of the cellular composition, and thus it could be argued that these cells are not meant to form a dominant population in the highly proliferative marrow tissue [47]. Additionally, if there is a maximal equilibrium for BMSC in the marrow, the existence of the murine BMSC population may function to further restrict the number of hBMSC that can be maintained simultaneously.

We quantified the relative number of hBMSC-Luc/GFP that were cycling (Ki-67^+^) or undergoing apoptosis (TUNEL) in non-irradiated and irradiated animals at 1 and 4 weeks, post-transplant. The number of cycling hBMSC-Luc/GFP was 20.3% ± 14.1% in irradiated animals 1-week after irradiation, while the number of cycling hBMSC-Luc/GFP was 2.4% ± 2.4% in non-irradiated animals. By contrast, there was a trend toward a higher rate of hBMSC-Luc/GFP apoptosis (TUNEL^+^) in animals that had not been irradiated. These data also suggest that irradiation gave the hBMSC-Luc/GFP a transient proliferative advantage in the mouse marrow, but that hBMSC viability declined as murine hematopoiesis recovered.

Limitations of this study include not tracking the clonal distribution of the hBMSC and not comparing hBMSC outcomes with an equivalent murine cell population. Viral bar coding of cells, to facilitate clonal tracking, is increasingly feasible [48], but remains costly. Future studies might consider using viral barcoding of transplanted cells to determine if specific clones are persisting in animals following transplant, and to identify the unique characteristics of the cells that do persist. This would also potentially allow investigators to determine if the clusters of cells observed in distal bone marrow cavities were clonally related. It would be useful to understand if a murine BMSC population would behave similarly to the hBMSC populations. A useful comparison with our study is a publication where a highly proliferative mouse BMSC clone, derived from the BMC-9 cell line, was transplanted into syngeneic irradiated mice [49]. The competitive mouse BMSC clone homed to sites of injury. Unlike our hBMSC-Luc/GFP, the mouse BMSC proliferated over time in the recipient mouse marrow, could be isolated, and serially transplanted in subsequent mice. The difference between observations reported by Lin et al. using mouse BMSC, and our observations with hBMSC likely reflect the reduced competitiveness of the hBMSC relative to more proliferative murine clones. Unlike the poliferative mouse BMSC clone [49], or highly proliferative human hematopoietic stem and progenitor cells [1], hBMSC do not appear capable of maintaining a high population within the mouse bone marrow cavitiy.

## Conclusion

This study provides a number of important insights relevant to the use of hBMSC in direct bone marrow injection models. First, this study is the first to demonstrate that it is possible to establish a high hBMSC content within an existing marrow cavity of a mouse, but that the number of hBMSC within the injected marrow cavity declines rapidly over time. While conditioning mice with sub-lethal irradiation enhances the persistence of the injected hBMSC in the short-term, hBMSC numbers still declined over time, especially following hematopoietic recovery in the mouse marrow. In conclusion, for short-term models (1–2 weeks) a high density of hBMSC can be established in murine marrow via direct bone marrow injection. For longer-term models (> 2 weeks), researchers should consider if the sparse population of the murine marrow with hBMSC will suffice. If this model is to be utilized, the temporal changes in murine bone marrow hBMSC content should be considered in experimental design and analysis.

## Supplementary Information

**Additional file 1: Supplementary Figure 1.** Flow cytometry analysis and tri-lineage differentiation of hBMSC from donors 1 and 2. **(A)** Flow cytometry characterization for donor 1. **(B)** Tri-lineage analysis for donor 1, demonstrating adipogenic (Oil Red O stained lipid vacuoles), osteogenic (Alizarin Red S stained mineralized matrix), and chondrogenic (Alcian Blue stained matrix) potential. **(C)** Flow cytometry phenotype characterization for donor 2. **(D)** Tri-lineage analysis for donor 2. In flow cytometry histograms, isotypes control staining is represented by shaded gray peaks, and stained hBMSC are represented with a black line. hBMSC were positive for CD73, CD90, CD105, CD44, CD146, and negative for CD31, CD34, CD45, and HLA-DR. Scale bars = 500 μm. **Supplementary Figure 2.** Characterization of donor 1 transduced hBMSC-Luc/GFP at passage 4. **(A)** hBMSC-Luc/GFP retained spindle-like morphology, and **(B)** were GFP^+^ (scale bar 100 μm). **(C)** Tri-lineage differentiation capacity of hBMSC-Luc/GFP was demonstrated by the positive formation of a cartilage-like matrix (Alcian Blue, scale bar 100 μm), **(D)** mineral deposits indicative of osteogenic tissue (Alizarin Red stain, scale bar 100 μm), and **(E)** oil droplet formation indicative of adipogenesis (Oil Red O stain, scale bar 50 μm). **(F)** Flow cytometry analysis demonstrated that hBMSC-Luc/GFP (green peak) were positive for GFP, CD90, CD105, CD44, CD146, CD73 and negative for CD45. Isotype staining is in shaded gray peaks. (**G and H**) Titration of hBMSC-Luc/GFP demonstrated that luciferase activity and resultant bioluminescence was linearly proportional to cell number. Error bars represent the standard deviation of each point. Statistical analysis using a Pearson co-efficient demonstrated a linear relationship between cell number and bioluminescence. **Supplementary Figure 3.** Characterization of donor 2 transduced hBMSC-Luc/GFP at passage 4. (**A**) hBMSC-Luc/GFP retained spindle-like morphology, and (**B**), were GFP+ (scale bar 100 μm). (**C**) Tri-lineage differentiation capacity of hBMSC-Luc/GFP was demonstrated by the positive formation of a cartilage-like matrix (Alcian Blue, scale bar 100 μm), (**D**) mineral deposits indicative of osteogenic tissue (Alizarin Red stain, scale bar 100 μm), and (**E**) oil droplet formation indicative of adipogenesis (Oil Red O stain, scale bar 50 μm). (**F**) Flow cytometry analysis demonstrated that hBMSC-Luc/GFP (green peak) were positive for GFP, CD90, CD105, CD44, CD146, CD73 and negative for CD45. Isotype staining is in shaded gray peaks. (**G and H**) Titration of hBMSC-Luc/GFP demonstrated that luciferase activity and resultant bioluminescence was linearly proportional to cell number. Error bars represent the standard deviation of each point Statistical analysis using a Pearson co-efficient demonstrated a linear relationship between cell number and bioluminescence. **Supplementary Figure 4. (A)** Bioluminscence emission from each Donor over time, Non-irradiated in blue, irradiated in red.. Donor 1 in triangles (Non-irradiated *n* = 7, Irradiated *n* = 9), Donor 2 in circles (Non-irradiated n = 9, Irradiated *n* = 11). Horizontal bar represents group average. (**B)**. Regression curves were fit to the data from combined donor results of Non-irradiated vs 2 Gy irradiated mice. Using Akaike’s Information Criterion (AICc) test in GraphPad, it was predicted with 99.92%) certainty that these data were better fit by the two curves, rather than by a single curve. **Supplementary Figure 5.** Comparison of hBMSC-Luc/GFP persistence in the femurs of male and female mice with and without 2 Gy sub-lethal irradiation prior to transplant. Figure provides a graphical display of the bioluminescence data for each animal across the 4-week study. Horizontal bar represents group average. In the Non-irradiated group there were 8 female and 8 males, and in the irradiated group there were 8 females and 12 males. **Supplementary Figure 6.** Analysis of hBMSC-Luc/GFP engraftment in mouse bones at week 4, post-transplant. **(A)** Bone marrow section demonstrating hBMSC-Luc/GFP cells (green) with complete cytoplasm staining while myeloid cells (based on nuclear morphology, marked with a white asterisk) remained unstained. Nuclear stain (blue) Scale bar 20 μm. Representative images of histology hBMSC-Luc/GFP detected in injected femur, lateral tibia and contralateral femur and tibia in (B) no cell mice, (C) non-irradiated and (D) 2 Gy irradiated mice. Scale bar 20 μm. **Supplementary Figure 7. (A)** hBMSC-Luc/GFP were associated with the bone (dotted white line), scale bar = 20 μm, **(B)** but not specifically with the sinusoidal vessels (yellow line) or with the elongated arterioles (dotted red line) within the bone marrow, scale bar = 20 μm. **(C)** The endosteal region was defined as ~ 13 cell diameters or 70 μm (blue line) from the edge of the bone Scale bar = 100 μm, inset scale bar = 20 μm. Bone width was determined to be ~ 700 μm wide using 90° andgle from bone. **Supplementary Table 1.** Antibodies used in experiments. **Supplementary Table 2.** Summary of hBMSC-Luc/GFP in histological sections of femurs and tibias. **Supplementary Table 3.** Cellularity of blood after sub-lethal irradiation.

## Data Availability

The datasets used and/or analyzed during the current study are available from the corresponding author upon reasonable request.

## Abbreviations

7-AAD: 7-amino-actinomycin D
AICc: Akaike’s Information Criterion
DMEM: Dulbecco’s modified Eagle’s medium
FBS: Fetal bovine serum
FGF-1: Fibroblast growth factor-1
GAG: Glycosaminoglycans
GFP: Green fluorescent protein
Gy: Gray
hBMSC-Luc/GFP: Human bone marrow-derived stromal cells that express luciferase and green fluorescent protein
hBMSC: Human BMSC
hBMSC: Human bone marrow-derived stromal cells
HSPC: Hematopeotic stem progenitor cell
IVIS: In Vivo Imaging System
Luc: Luciferase
MSCV: Murine Stem Cell Virus
NOD: Non-obese diabetic
NSG: NOD-*scid* interleukin (IL)-2γ^−/−^
PFA: Paraformaldehyde
*scid*: Severe combined immune deficient
QUT: Queensland University of Technology
