# Supplementary material for: Human bone marrow-derived stromal cell behavior when injected directly into the bone marrow of NOD-scid-gamma mice pre-conditioned with sub-lethal irradiation
Source: Stem Cell Res Ther. 2021 Apr 12;12:231. doi: 10.1186/s13287-021-02297-7 (PMC8042930; doi:10.1186/s13287-021-02297-7)
Supplement: Supplementary file 1 — Additional file 1: Supplementary Figure 1. Flow cytometry analysis and tri-lineage differentiation of hBMSC from donors 1 and 2. (A) Flow cytometry characterization for donor 1. (B) Tri-lineage analysis for donor 1, demonstrating adipogenic (Oil Red O stained lipid vacuoles), osteogenic (Alizarin Red S stained mineralized matrix), and chondrogenic (Alcian Blue stained matrix) potential. (C) Flow cytometry phenotype characterization for donor 2. (D) Tri-lineage analysis for donor 2. In flow cytometry histograms, isotypes control staining is represented by shaded gray peaks, and stained hBMSC are represented with a black line. hBMSC were positive for CD73, CD90, CD105, CD44, CD146, and negative for CD31, CD34, CD45, and HLA-DR. Scale bars = 500 μm. Supplementary Figure 2. Characterization of donor 1 transduced hBMSC-Luc/GFP at passage 4. (A) hBMSC-Luc/GFP retained spindle-like morphology, and (B) were GFP+ (scale bar 100 μm). (C) Tri-lineage differentiation capacity of hBMSC-Luc/GFP was demonstrated by the positive formation of a cartilage-like matrix (Alcian Blue, scale bar 100 μm), (D) mineral deposits indicative of osteogenic tissue (Alizarin Red stain, scale bar 100 μm), and (E) oil droplet formation indicative of adipogenesis (Oil Red O stain, scale bar 50 μm). (F) Flow cytometry analysis demonstrated that hBMSC-Luc/GFP (green peak) were positive for GFP, CD90, CD105, CD44, CD146, CD73 and negative for CD45. Isotype staining is in shaded gray peaks. (G and H) Titration of hBMSC-Luc/GFP demonstrated that luciferase activity and resultant bioluminescence was linearly proportional to cell number. Error bars represent the standard deviation of each point. Statistical analysis using a Pearson co-efficient demonstrated a linear relationship between cell number and bioluminescence. Supplementary Figure 3. Characterization of donor 2 transduced hBMSC-Luc/GFP at passage 4. (A) hBMSC-Luc/GFP retained spindle-like morphology, and (B), were GFP+ (scale bar 100 μm). (C) Tri-l [file 13287_2021_2297_MOESM1_ESM.docx]

## Supplementary Tables

**Supplementary Table 1.** Antibodies used in experiments.

| **Antibody** | **Catalogue number** | **Company** | **Concentration used** |
| --- | --- | --- | --- |
| **Flow cytometry antibodies** |  |  |  |
| Hu CD90 BV421 | 328122 | Biolegend | 1 test |
| Hu CD45 BV421 | 304032 | Biolegend | 1 test |
| Hu CD146 APC | 130-092-849 | Miltenyi Bioscience | 2.5 tests |
| Hu CD73 APC | 130-095-183 | Miltenyi Bioscience | 2.5 tests |
| Hu CD44 PE | 130-095-180 | Miltenyi Bioscience | 2.5 tests |
| Hu CD271 PE | 130-091-885 | Miltenyi Bioscience | 2.5 tests |
| Hu CD105 PE | 130-094-941 | Miltenyi Bioscience | 2.5 tests |
| Hu CD34 (Mouse IgG2a) | 130-090-954 | Miltenyi Bioscience | 1 test |
| Hu HLA-DR (IgG2a) | 130-095-298 | Miltenyi Bioscience | 1 test |
| Mu CD45 APC | 559864 | Beckman Dickson | 1 µg/mL |
| Hu/mu CD11b PE | 101208 | Biolegend | 1 µg/mL |
| Mu Gr-1 (Ly6G/C) APCCy7 | 557661 | Beckman Dickson | 1 µg/mL |
| Fc Block | 553142 | Beckman Dickson | 0.1 µg/mL |
| **IHC/F antibodies** |  |  |  |
| Chicken anti-GFP | Ab13970 | Abcam | 40 µg/mL |
| Donkey anti-Chicken A647 | 703-065-155 | Jackson Immunology | 3 µg/mL |
| Goat anti-Rabbit A546 | A11012 | Life Technologies | 4 µg/mL |
| Rabbit anti-Ki-67 | 5278384001 | Roche Healthcare | 2 µg/mL |
| **Isotype controls** |  |  |  |
| Mouse IgG_1_ PE | 130-092-212 | Miltenyi Bioscience | 2.5 tests |
| Mouse IgG_1_ APC | 130-092-214 | Miltenyi Bioscience | 2.5 tests |
| Mouse IgG_1_ BV421 | 400158 | Biolegend | 1 test |
| Mouse IgG_2a_ BV421 | 400259 | Biolegend | 1 test |

Acronyms: APC, Allophycocyanin; A546, Alexa Fluor 546; A647, Alexa Fluor 647; BV, Brilliant Violet; Cy7, cyanine dye -7; GFP, green fluorescent protein; h, Human; m, mouse; PE, R-phycoerythrin.

**Supplementary Table 2.** Summary of hBMSC-Luc/GFP in histological sections of femurs and tibias.

|  |  | Injected femur | Lateral tibia | Contra-lateral femur | Contra-lateral tibia | Treatment average | Overall average |
| --- | --- | --- | --- | --- | --- | --- | --- |
| Per cent of total GFP in bone marrow (DAPI cells) | Non-irradiated | 3.3 ± 2.5% | 0.6 ± 0.7% | 0.7 ± 1.1% | 2.0 ± 1.8% | 1.3 ± 1.1% | 1.8 ± 1.3% |
|  | Irradiated | 3.9 ± 2.6% | 2.1 ± 1.7% | 1.8 ± 1.8% | 2.2 ± 2.2% | 2.2 ± 1.4% |  |
| Per cent of GFP near endosteal (within 70 µm of bone) | Non-irradiated | 51.9 ± 11.3% | 49.5 ± 8.8% | 48.8 ± 29.7% | 68.6 ± 9.1% | 52.6 ± 16.7% | 54.5 ± 12.4% |
|  | Irradiated | 47.5 ± 7.7% | 57.0 ± 22.2% | 61.5 ± 18.3% | 59.8 ± 24.0% | 55.6 ± 9.5% |  |
| GFP cells within 3 cell diameters | Non-irradiated | 83.2 ± 8.0% | 51.0 ± 30.0% | 64.1 ± 12.3% | 70.2 ± 28.8% | 60.1 ± 27.3% | 66.1 ± 20.6% |
|  | Irradiated | 76.5 ± 15.8% | 67.4 ± 27.1% | 68.8 ± 17.6% | 78.5 ± 6.4% | 69.8 ± 15.4% |  |

Results shown as average percent (%) of total nucleated cells ± standard deviation. Acronyms: GFP, green fluorescent protein; DAPI, 4′, 6-diamidino-2-phenylindole

**Supplementary Table 3.** Cellularity of blood after sub-lethal irradiation.

|  | **Day 3** |  |  | **Day 7** |  |  | **Day 10** |  |  |
| --- | --- | --- | --- | --- | --- | --- | --- | --- | --- |
| **Parameter** | **Non-Irradiated** | **Irradiated** | **Statistics** | **Non-Irradiated** | **Irradiated** | **Statistics** | **Non-Irradiated** | **Irradiated** | **Statistics** |
| WBC/mL | 1.80 x10^6^ ± 0.59x10^6^ | 0.56 x10^6^ ± 0.17 x10^6^ | * p=0.0157 | 3.90 x10^6^ ± 0.82x10^6^ | 1.73 x10^6^ ± 0.48 x10^6^ | ** p=0.0044 | 2.75 x10^6^ ± 0.12x10^6^ | 2.10 x10^6^ ± 0.60 x10^6^ | ns p=>0.9999 |
| Platelets /mL | 1.30 x10^9^ ± 0.14 x10^9^ | 0.80 x10^9^ ± 0.11 x10^9^ | *** p=0.0001 | 1.32 x10^9^ ± 0.19 x10^9^ | 0.21 x10^9^ ± 0.02 x10^9^ | **** p=<0.0001 | 1.12 x10^9^ ± 0.01 x10^9^ | 0.34 x10^9^ ± 0.01 x10^9^ | **** p=<0.0001 |
| RBC/mL | 7.00 x10^9^± 0.44 x10^9^ | 6.08 x10^9^ ± 0.66x10^9^ | ns p=0.1584 | 7.88 x10^9^± 0.59 x10^9^ | 6.68 x10^9^ ± 0.86 x10^9^ | ns p=0.1584 | 7.47 x10^9^ ± 0.37 x10^9^ | 5.89 x10^9^ ± 0.73 x10^9^ | * p=0.0216 |
| haemoglobin g/dL | 1.31 x 10^2^ ± 0.09 x10^2^ | 1.14 x 10^2^ ± 0.13 x 10^2^ | ns p=0.1686 | 1.47 x 10^2^ ± 0.01 x 10^2^ | 1.23 x 10^2^ ± 0.16 x 10^2^ | ns p=0.1024 | 1.40 x 10^2^ ± 0.00 x 10^2^ | 1.12 x 10^2^ ± 0.01 x 10^2^ | * p=0.0177 |
| CD45^+^ 7-AAD^-^ /mL | 4.40 x 10^5^ ± 2.51 x 10^5^ | 4.61 x 10^5^ ± 0.04 x 10^3^ | * p=0.0140 | 6.10 x 10^5^ ± 3.76x10^5^ | 0.71 x 10^5^ ± 0.39 x 10^5^ | * p=0.351 | 1.05 x 10^5^ ± 1.26 x 10^5^ | 1.98 x 10^5^ ± 2.21 x 10^5^ | ns p=0.8525 |
| CD45^+^ 7-AAD^-^ CD11b^+^ Gr^-^1^+^ /mL | 3.90 x 10^5^ ± 2.25 x 10^5^ | 0.01 x 10^5^ ± 0.01 x 10^4^ | * p=0.0143 | 5.03 x 10^5^ ± 0.33x10^5^ | 0.60 x 10^5^ ± 0.33 x 10^5^ | p= 0.0291 | 0.93 x 10^5^ ± 1.1 x 10^5^ | 1.79 x 10^5^ ± 1.98 x 10^5^ | ns p=0.8434 |
| CD45^+^ 7-AAD^-^ CD11b^+^ Gr^-^1^low^ /mL | 4.46 x 10^4^ ± 2.4 x 10^4^ | 0.27 x 10^5^ ± 0.28 x 10^4^ | p=0.0155 | 9.66 x 10^4^ ± 8.04x10^4^ | 1.02 x 10^4^ ± 0.66 x 10^4^ | ns p=0.1272 | 0.95 x 10^4^ ± 1.12 x 10^4^ | 1.70 x 10^4^ ± 2.27 x 10^4^ | ns p=0.9801 |

Statistics: using multiple t-test with multiple comparison utilising the Holm-Sidak’s correction. ns, not significant; * p ≤ 0.05, ** p ≤ 0.01, *** p ≤ 0.001, **** p ≤ 0.0001.Acronyms: RBC, red blood cells; WBC, white blood cells;


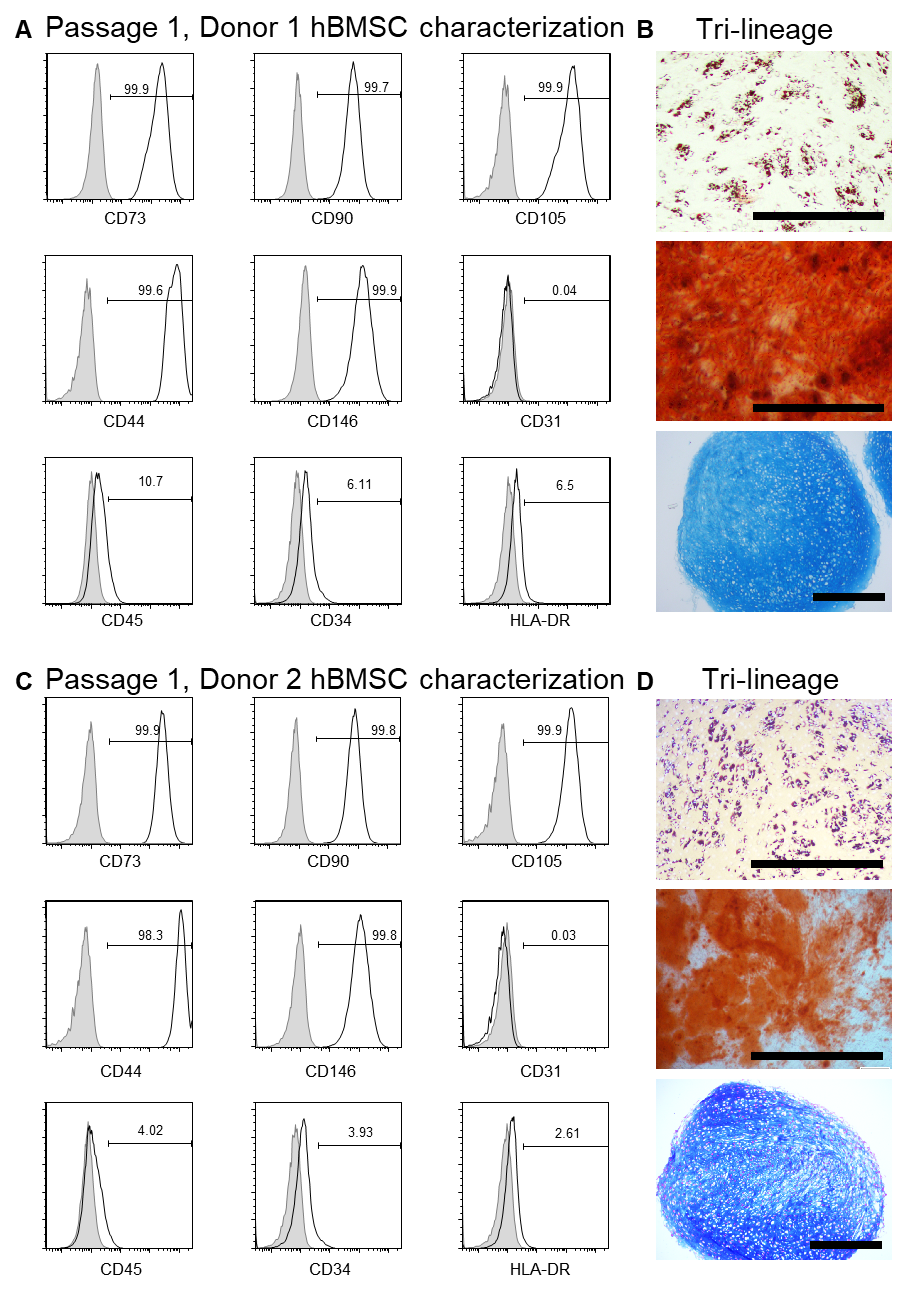


**Supplementary Figure** 1. Flow cytometry analysis and tri-lineage differentiation of hBMSC from donors 1 and 2. **(A)** Flow cytometry characterization for donor 1. **(B)** Tri-lineage analysis for donor 1, demonstrating adipogenic (Oil Red O stained lipid vacuoles), osteogenic (Alizarin Red S stained mineralized matrix), and chondrogenic (Alcian Blue stained matrix) potential. **(C)** Flow cytometry phenotype characterization for donor 2. **(D)** Tri-lineage analysis for donor 2. In flow cytometry histograms, isotypes control staining is represented by shaded grey peaks, and stained hBMSC are represented with a black line. hBMSC were positive for CD73, CD90, CD105, CD44, CD146, and negative for CD31, CD34, CD45, and HLA-DR. Scale bars = 500 µm.


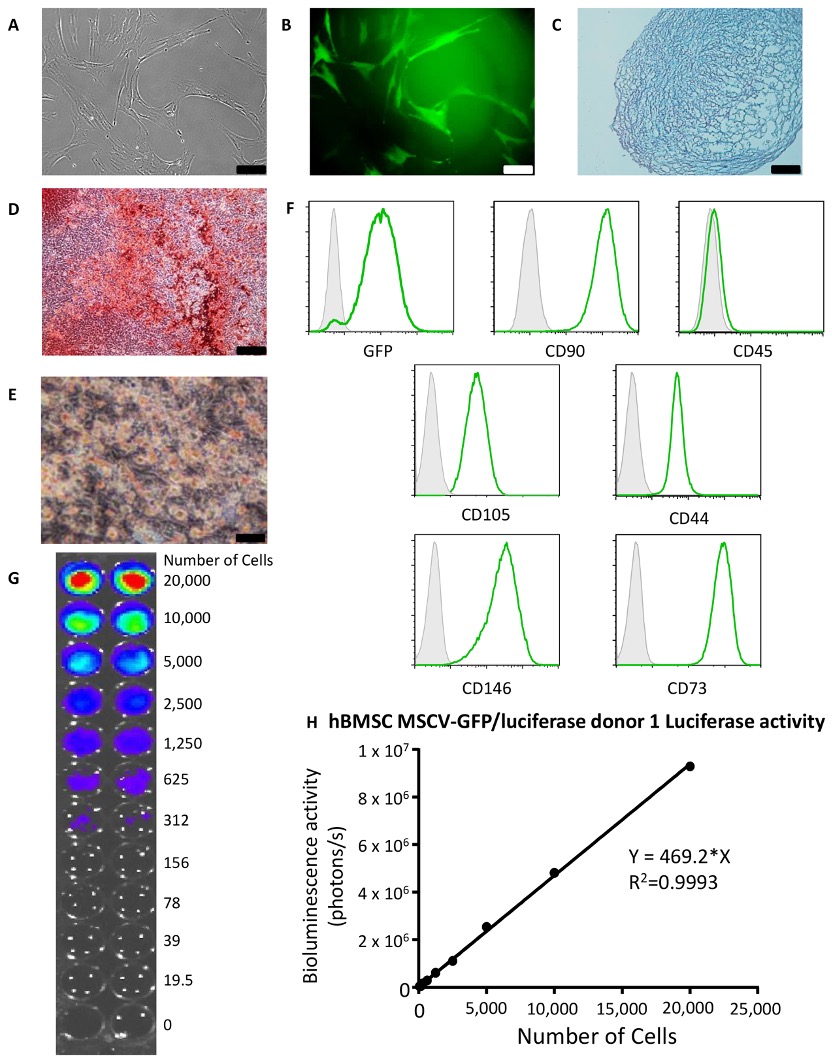


**Supplementary Figure** **2**. Characterization of donor 1 transduced hBMSC-Luc/GFP at passage 4. **(A)** hBMSC-Luc/GFP retained spindle-like morphology, and **(B)** were GFP^+^ (scale bar 100 μm). **(C)** Tri-lineage differentiation capacity of hBMSC-Luc/GFP was demonstrated by the positive formation of a cartilage-like matrix (Alcian Blue, scale bar 100 μm), **(D)** mineral deposits indicative of osteogenic tissue (Alizarin Red stain, scale bar 100 μm), and **(E)** oil droplet formation indicative of adipogenesis (Oil Red O stain, scale bar 50 μm). **(F)** Flow cytometry analysis demonstrated that hBMSC-Luc/GFP (green peak) were positive for GFP, CD90, CD105, CD44, CD146, CD73 and negative for CD45. Isotype staining is in shaded grey peaks. (**G and H**) Titration of hBMSC-Luc/GFP demonstrated that luciferase activity and resultant bioluminescence was linearly proportional to cell number. Error bars represent the standard deviation of each point. Statistical analysis using a Pearson co-efficient demonstrated a linear relationship between cell number and bioluminescence.


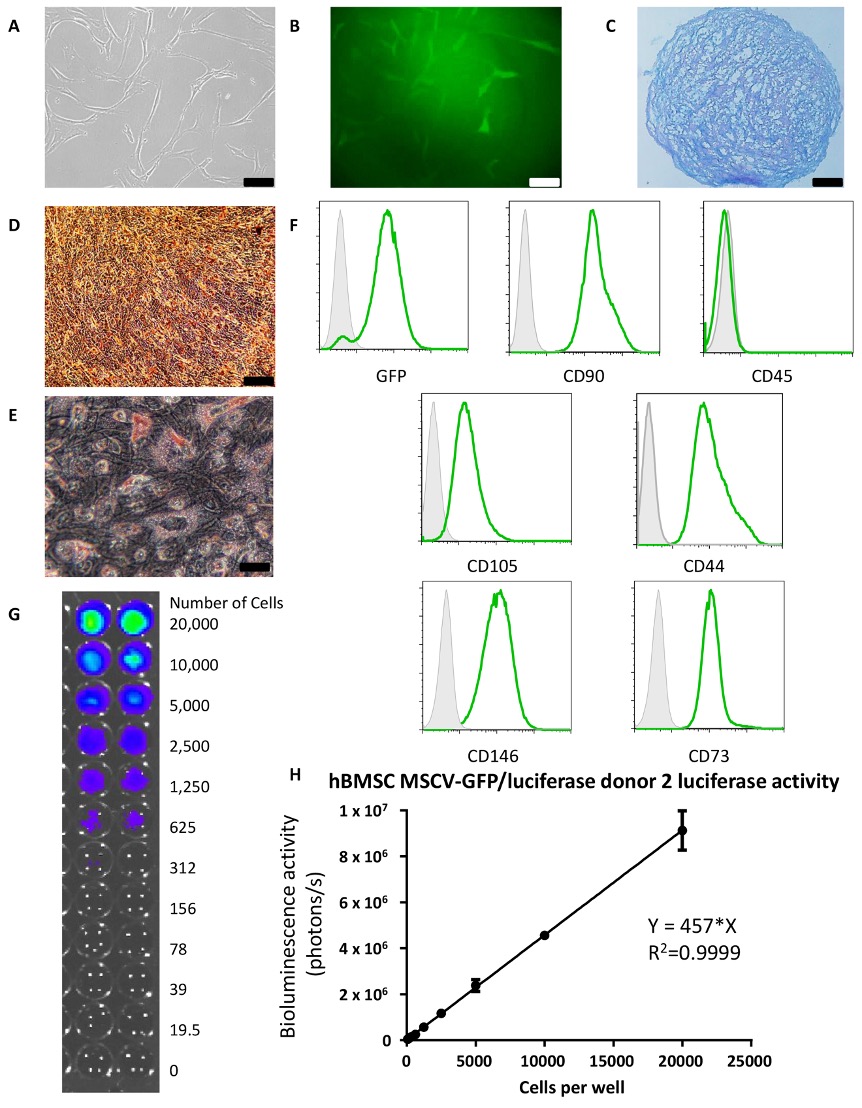


**Supplementary Figure 3.** Characterization of donor 2 transduced hBMSC-Luc/GFP at passage 4. (**A**) hBMSC-Luc/GFP retained spindle-like morphology, and (**B**), were GFP+ (scale bar 100 μm). (**C**) Tri-lineage differentiation capacity of hBMSC-Luc/GFP was demonstrated by the positive formation of a cartilage-like matrix (Alcian Blue, scale bar 100 μm), (**D**) mineral deposits indicative of osteogenic tissue (Alizarin Red stain, scale bar 100 μm), and (**E**) oil droplet formation indicative of adipogenesis (Oil Red O stain, scale bar 50 μm). (**F**) Flow cytometry analysis demonstrated that hBMSC-Luc/GFP (green peak) were positive for GFP, CD90, CD105, CD44, CD146, CD73 and negative for CD45. Isotype staining is in shaded grey peaks. (**G and H**) Titration of hBMSC-Luc/GFP demonstrated that luciferase activity and resultant bioluminescence was linearly proportional to cell number. Error bars represent the standard deviation of each point Statistical analysis using a Pearson co-efficient demonstrated a linear relationship between cell number and bioluminescence.


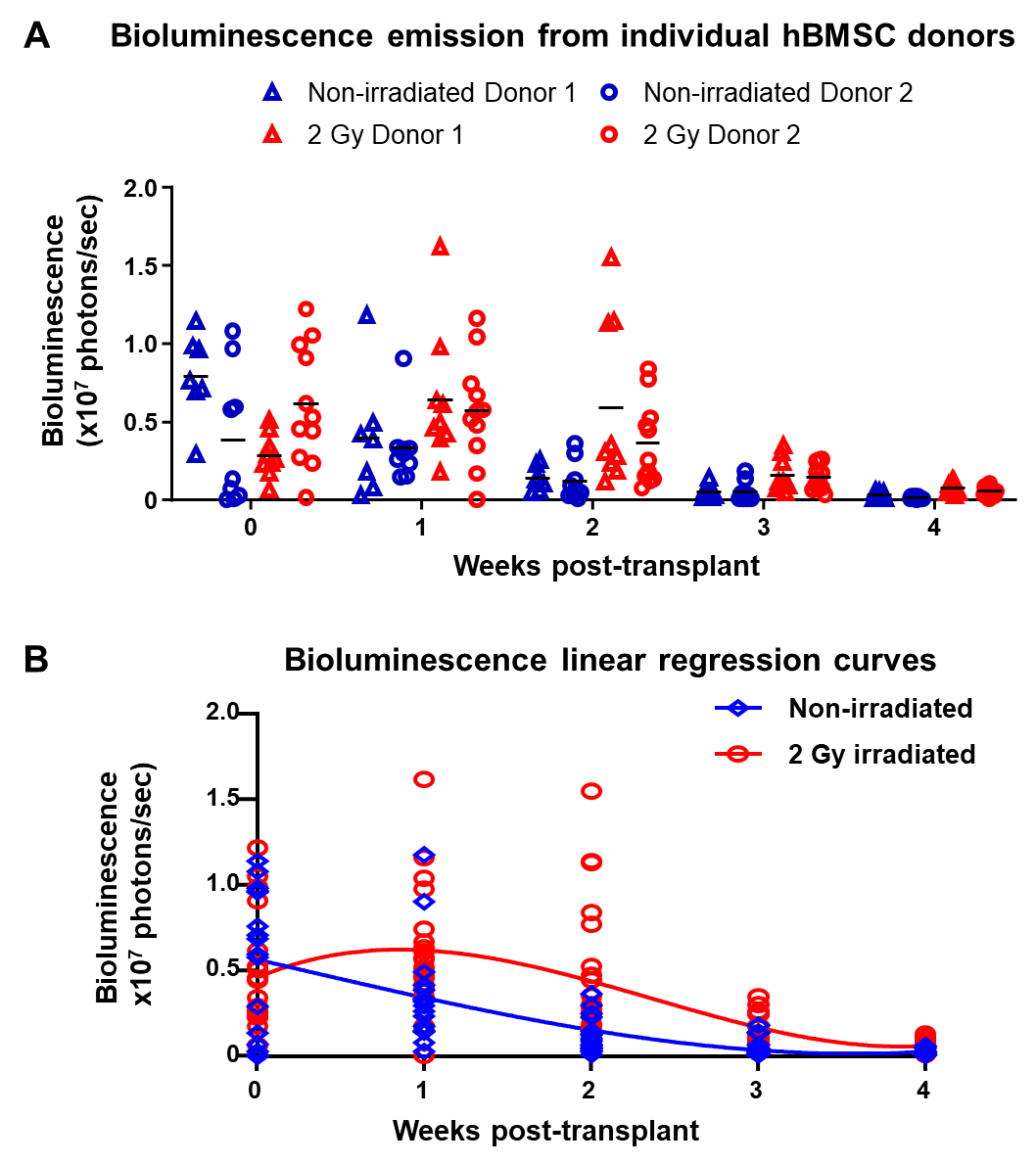


**Supplementary Figure 4. (A)** Bioluminscence emission from each Donor over time, Non-irradiated in blue, irradiated in red. . Donor 1 in triangles (Non-irradiated n=7, Irradiated n=9), Donor 2 in circles (Non-irradiated n=9, Irradiated n=11).Horizontal bar represents group average. (**B)**. Regression curves were fit to the data from combined donor results of Non-irradiated vs 2 Gy irradiated mice. Using Akaike’s Information Criterion (AICc) test in GraphPad, it was predicted with 99.92%) certainty that these data were better fit by the two curves, rather than by a single curve.


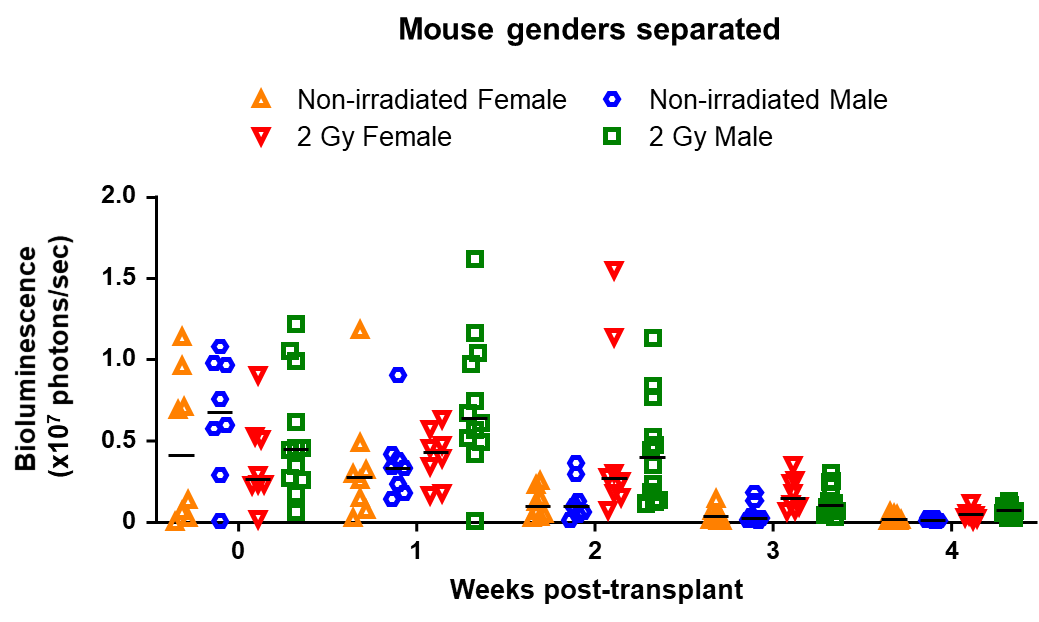


**Supplementary Figure 5.** Comparison of hBMSC-Luc/GFP persistence in the femurs of male and female mice with and without 2 Gy sub-lethal irradiation prior to transplant. Figure provides a graphical display of the bioluminescence data for each animal across the 4-week study. Horizontal bar represents group average. In the Non-irradiated group there were 8 female and 8 males, and in the irradiated group there were 8 females and 12 males.


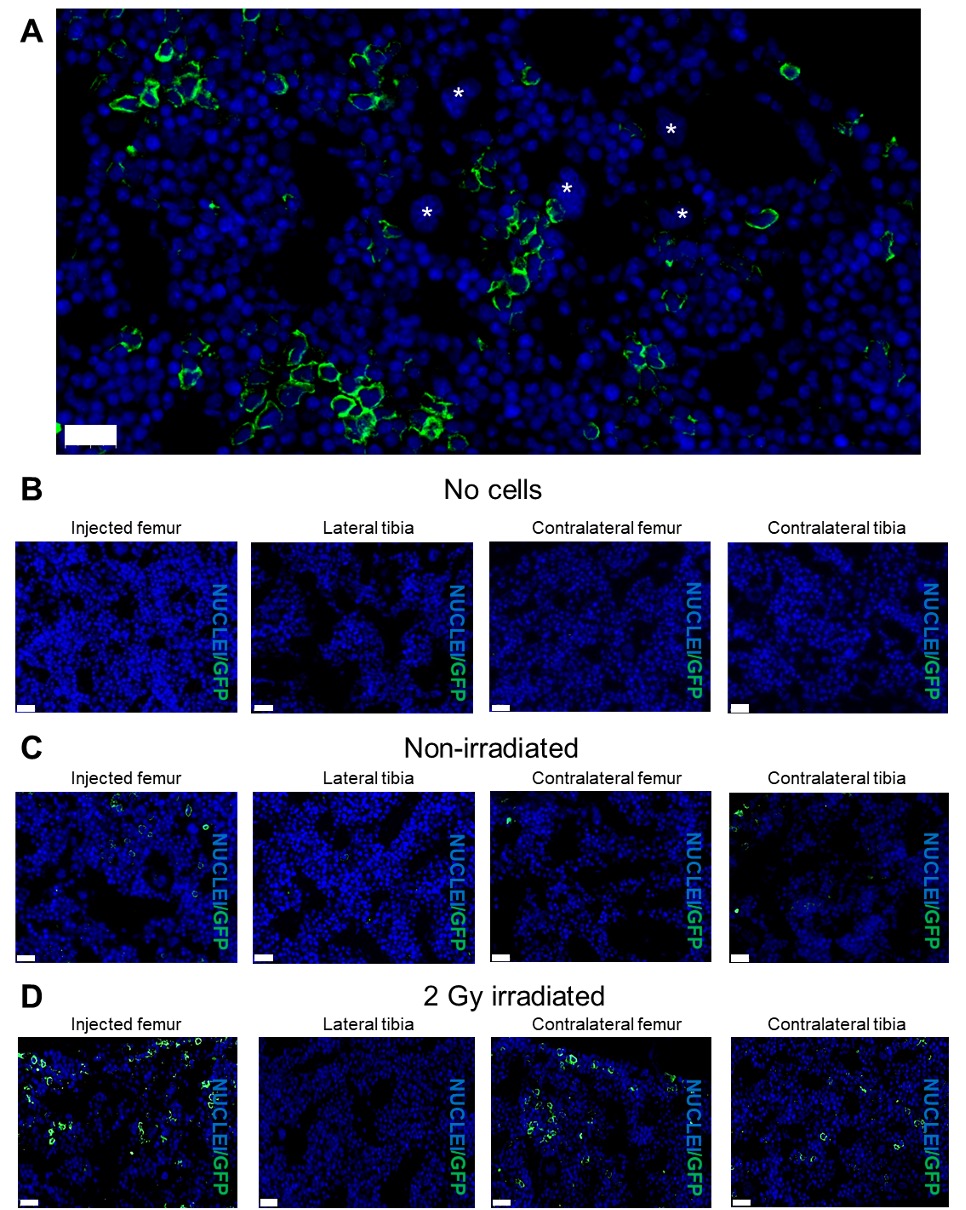


**Supplementary Figure 6.** Analysis of hBMSC-Luc/GFP engraftment in mouse bones at week 4, post-transplant. **(A)** Bone marrow section demonstrating hBMSC-Luc/GFP cells (green) with complete cytoplasm staining while myeloid cells (based on nuclear morphology, marked with a white asterisk) remained unstained. Nuclear stain (blue) Scale bar 20 µm. Representative images of histology hBMSC-Luc/GFP detected in injected femur, lateral tibia and contralateral femur and tibia in (B) no cell mice, (C) non-irradiated and (D) 2 Gy irradiated mice. Scale bar 20 µm.


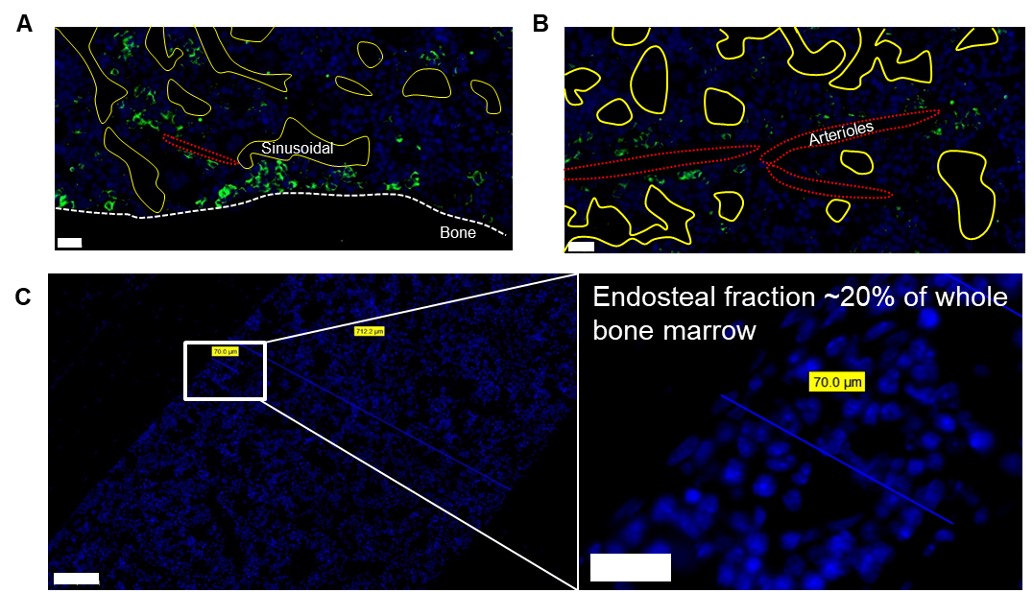


**Supplementary Figure 7** **(A)** hBMSC-Luc/GFP were associated with the bone (dotted white line), scale bar = 20 µm, **(B)** but not specifically with the sinusoidal vessels (yellow line) or with the elongated arterioles (dotted red line) within the bone marrow, scale bar = 20 µm. **(C)** The endosteal region was defined as ~13 cell diameters or 70 µm (blue line) from the edge of the bone Scale bar = 100 µm, inset scale bar = 20 µm. Bone width was determined to be ~700 µm wide using 90° andgle from bone.
